# DEMENTIA WITHOUT BORDERS: BUILDING COMMUNITY CONNECTIONS TO REDUCE STIGMA AND FOSTER INCLUSION

**DOI:** 10.1093/geroni/igz038.3453

**Published:** 2019-11-08

**Authors:** Alison Phinney, Marigrace Becker, Lee Burnside, Gloria Puurveen

**Affiliations:** 1 University of British Columbia, Vancouver, British Columbia, Canada; 2 University of Washington, Seattle, Washington, United States

## Abstract

BACKGROUND: The concept of social citizenship is gaining traction in the field of dementia studies, but as a practical tool to guide development of supports and services, it remains poorly understood. A one year project to promote collaboration between University of Washington in Seattle and University of British Columbia in Vancouver addressed this very question. Activities were undertaken so these communities could know each other better, with researchers, service providers and people with dementia connecting to share knowledge and expertise. PURPOSE: The project culminated with a public festival to put into practice and share some of what was learned over the year. METHODS: People with dementia and care partners helped plan “Dementia Without Borders”, held at an international park straddling the border between Seattle and Vancouver. 150 people came from the US and Canada, including many people with dementia, family members and friends. The day began with a community walk and gift exchange, followed by a meal and creative activities including poetry readings, music, an art exhibit, and quilt making. RESULTS: Evaluation was overwhelmingly positive with people expressing a sense of hope and belonging. For some, it was their first time to speak openly about having dementia, and meeting others in this space was a joy-filled experience. CONCLUSIONS: This project has leveraged the symbolic power of an international border to raise awareness of the importance of social connection for people with dementia. We further explore how the notion of “dementia without borders” extends theoretical and practical understanding of social citizenship.

